# Digital Gamification Tools to Enhance Vaccine Uptake: Scoping Review

**DOI:** 10.2196/47257

**Published:** 2024-02-29

**Authors:** Hina Hakim, S Michelle Driedger, Dominique Gagnon, Julien Chevrier, Geneviève Roch, Eve Dubé, Holly O Witteman

**Affiliations:** 1 Department of Family and Emergency Medicine Université Laval Québec City, QC Canada; 2 Department of Community Health Sciences University of Manitoba Winnipeg, MB Canada; 3 Direction des risques biologiques Institut national de santé publique du Québec Quebec City, QC Canada; 4 Bibliothèque Louise-Lalonde-Lamarre Polytechnique Montréal Montréal, QC Canada; 5 Faculty of Nursing Université Laval Quebec City, QC Canada; 6 Centre hospitalier universitaire (CHU) de Québec-Université Laval Université Laval Quebec City, QC Canada; 7 VITAM Research Centre for Sustainable Health Université Laval Quebec City, QC Canada; 8 Département d’anthropologie Université Laval Quebec City, QC Canada

**Keywords:** digital gamified tools, digital game, vaccination, gamification, vaccine uptake, scoping review, review method, vaccine, gamified, COVID-19, COVID, SARS-CoV-2, health behaviour, health behavior, health promotion, behavior change, behaviour change

## Abstract

**Background:**

Gamification has been used successfully to promote various desired health behaviors. Previous studies have used gamification to achieve desired health behaviors or facilitate their learning about health.

**Objective:**

In this scoping review, we aimed to describe digital gamified tools that have been implemented or evaluated across various populations to encourage vaccination, as well as any reported effects of identified tools.

**Methods:**

We searched Medline, Embase, CINAHL, the Web of Science Core Collection, the Cochrane Database of Systematic Reviews, the Cochrane Central Register of Controlled Trials, Academic Search Premier, PsycInfo, Global Health, and ERIC for peer-reviewed papers describing digital gamified tools with or without evaluations. We also conducted web searches with Google to identify digital gamified tools lacking associated publications. We consulted 12 experts in the field of gamification and health behavior to identify any papers or tools we might have missed. We extracted data about the target population of the tools, the interventions themselves (eg, type of digital gamified tool platform, type of disease/vaccine, type and design of study), and any effects of evaluated tools, and we synthesized data narratively.

**Results:**

Of 1402 records, we included 28 (2%) peer-reviewed papers and 10 digital gamified tools lacking associated publications. The experts added 1 digital gamified tool that met the inclusion criteria. Our final data set therefore included 28 peer-reviewed papers and 11 digital gamified tools. Of the 28 peer-reviewed papers, 7 (25%) explained the development of the tool, 16 (57%) described evaluation, and 2 (7%) reported both development and evaluation of the tool. The 28 peer-reviewed papers reported on 25 different tools. Of these 25 digital gamified tools, 11 (44%) were web-based tools, 8 (32%) mobile (native mobile or mobile-enabled web) apps, and 6 (24%) virtual reality tools. Overall, tools that were evaluated showed increases in knowledge and intentions to receive vaccines, mixed effects on attitudes, and positive effects on beliefs. We did not observe discernible advantages of one type of digital gamified tool (web based, mobile, virtual reality) over the others. However, a few studies were randomized controlled trials, and publication bias may have led to such positive effects having a higher likelihood of appearing in the peer-reviewed literature.

**Conclusions:**

Digital gamified tools appear to have potential for improving vaccine uptake by fostering positive beliefs and increasing vaccine-related knowledge and intentions. Encouraging comparative studies of different features or different types of digital gamified tools could advance the field by identifying features or types of tools that yield more positive effects across populations and contexts. Further work in this area should seek to inform the implementation of gamification for vaccine acceptance and promote effective health communication, thus yielding meaningful health and social impacts.

## Introduction

Vaccination is one of the most cost-effective methods of preventing the spread of vaccine-preventable diseases. If vaccination coverage falls below the thresholds that are safe for the prevention of epidemic transmission, the incidence of vaccine-preventable diseases increases [1,2]. For example, measles returned over the past 2 decades, and the incidence of measles in the European Union increased in 2017-2018 [3].

In 2019, prior to the COVID-19 pandemic, the World Health Organization identified vaccine hesitancy (ie, the reluctance or refusal to be vaccinated despite the availability of vaccination services) as 1 of the top 10 threats to worldwide health [4]. Vaccine hesitancy is one of the several reasons some people are un- or undervaccinated [5-9]. Interventions addressing vaccine hesitancy are therefore necessary to promote vaccine acceptance and uptake. As the contributors of vaccine acceptance are diverse, no single intervention will solve this issue [10]. Multicomponent interventions tailored to local barriers to vaccine acceptance and uptake are known to be the most effective [11,12]. Misinformation and conspiracy theories spread online, where extensive antivaccine content is shared [13-15], potentially negatively influencing views about vaccines [16,17]. Efforts have been made to counter vaccine misinformation and mistrust by targeting various groups, such as parents, non–health care workers [18,19], and adolescents [20], and delivering information about the risks and benefits of different types of vaccines, for instance, human papillomavirus (HPV) vaccination [21] and measles, mumps, and rubella vaccines [22,23]. Along with traditional communication strategies, the use of other strategies to inform and educate about immunizations, for example, with digital gamified tools, may help encourage vaccine uptake.

Gamification is defined as the use of game design elements in nongame contexts [24]. It includes several aspects and features, such as fun interfaces, immediate success or feedback, reward systems (levels, point scores, badges), challenges and competitions, team playing, avatars, and quizzes. Previous studies have used gamification to achieve desired health behaviors [25-27] or facilitate their learning about health [28]. Gamification draws on elements from serious games, meaning fully developed digital games used to train and educate players [29,30]. For example, a serious game “Land of Secret Gardens” facilitates conversations about HPV with preteens. In the game, preteens need to protect their bodies with a “potion,” which offers a metaphor for the HPV vaccine [31]. However, serious games and digital gamified tools are distinct but related concepts. Serious games use gaming as a central and primary medium [32]. In contrast, digital gamified tools (eg, apps) or gamified interventions are not complete game experiences but have gaming features, such as rewards systems, scoring of points, or engaging users in different challenges [33]. In this study, we defined digital gamified tools as digital apps with the aforementioned gaming features. Our definition includes serious games that meet the criteria, that is, they must include such gaming features. This scoping review provides insight into the reported effects of digital gamified tools to increase vaccine uptake. Our review built upon existing reviews in the field by including a comprehensive search of both published literature and online tools, as well as an examination of both the characteristics and the reported effects of these digital tools. This review was distinct in that it focused specifically on digital gamified tools and their effects, rather than simply the effectiveness of gamification in general. In doing so, this review aimed to fill a gap in the literature by providing evidence-based answers to the question of whether gamification “works” to increase vaccine uptake. Therefore, the objectives of this scoping review [34] were, first, to review digital gamified tools that have been implemented or evaluated across various populations to encourage vaccine uptake and, second, to describe any reported effects of the identified tools in terms of influence on users’ knowledge or behavior toward vaccination. Our research questions can therefore be summarized as follows:

- What digital gamified tools intended to encourage vaccination exist and have been described in the literature?
- Do these tools demonstrate any effects on knowledge, attitudes, beliefs, and behaviors about vaccination?

## Methods

### Search Strategy

For peer-reviewed papers, we searched Medline (Ovid), Embase (Ovid), CINAHL (EBSCO), the Web of Science Core Collection, the Cochrane Database of Systematic Reviews (Ovid), the Cochrane Central Register of Controlled Trials (Ovid), Academic Search Premier (EBSCO), PsycInfo (Ovid), Global Health (Ovid), and ERIC (Ovid) with no language or date restrictions. The proposed search terms were, for example, “vaccine,” “vaccination,” “immunization,” “video games,” “gamification,” “application,” and “virtual reality” (see Multimedia Appendix 1 for the full search strategy). The search was conducted on January 26 and 27, 2022.

We also conducted an online Google search on May 5, 2022, for any digital tools with gamified features that deliver informative or educative messages on vaccination. The search terms we used were “vaccination,” “immunization,” “electronic game,” “computer game,” “mobile game,” “interactive game,” and “digital game” (see Multimedia Appendix 1 for the full search strategy). We reviewed the first 30 results for each search, as it is rare for users to click past the third page of 10 search results per page, and therefore, researchers analyzing medical content available on the web often use 30 as a threshold [35-37]. On May 6, 2022, we conducted the same searches in private browsing mode to ascertain whether our results had been affected by a “filter bubble” [38], that is, the way Google search results are adapted to one’s previous browsing activity. Our search strategy was constructed and reviewed by 2 librarians. Following the librarians’ advice, we expanded our search strategy to include ERIC and Global Health databases.

### Study Selection and Screening Process

We used PICO (Population, Intervention, Comparison, and Outcome) to structure study inclusion and exclusion criteria (Table 1). Our population of interest was the general public or any subgroup, including health care professionals and students. We sought studies describing tools with gamification techniques or gamified elements, including gamified web-based quizzes to deliver informative or educative messages on vaccination. Posters, preprints, editorials, conference proceedings, news bulletins, and paper-based or board games were excluded. Our comparator was any control, including offering no education on vaccination or comparing participants before and after an intervention. Our outcomes of interest included common outcomes associated with vaccine uptake, namely knowledge (comprehension, understanding), attitudes (for or against vaccination), beliefs (perceived benefits, perceived risks), and behaviors toward vaccines (vaccination intention [ie, intention to get vaccinated or not get vaccinated] and vaccine uptake [ie, receiving or not receiving a vaccine]). We excluded papers that did not present the description or evaluation of a concrete digital gamified tool.

**Table 1:** Inclusion and exclusion criteria.

Component	Inclusion criteria	Exclusion criteria	Question related to the criteria
Type of report	Original paper Evaluated intervention or digital gamified tool	Posters, preprints, and conference proceedings Modeling or simulation study Brochures Editorials Bulletins	Has the study or research described the development of the tool and evaluated it?
Population	General public (any subgroup) Professionals Students	N/A^a^	Who is the audience for whom the key message was intended?
Intervention	Tools with gamification technique or gamified elements, including gamified web-based quizzes to deliver informative or educative messages on vaccination	Any study or gamification tools not intended for vaccination/vaccine uptake Studies or apps to reduce vaccine pain and fears and to report immunization status or record keeping, surveillance or vaccine coverage apps, contact-tracing or early detection apps Paper games, board games (not digital) Videos with no gamified element included	Does the study or tool aim to deliver an informative or educative message on vaccination?
Comparator	Any control, including offering no education or no digital gamified tool	N/A	N/A
Outcome	Common outcomes that encourage vaccine uptake: knowledge (comprehension, understanding), attitudes (for or against vaccination, beliefs (risk perception, etc), behaviors toward vaccines (vaccination intention [ie, intention to get vaccinated or not get vaccinated] and vaccine uptake [ie, receiving or not receiving a vaccine])	Outcomes not related to the encouragement of vaccine uptake	Has the study or tool been evaluated for the outcomes that encourage vaccine uptake?

^a^N/A: not applicable.

For Google-searched digital gamified tools, our inclusion and exclusion criteria used the same specifications regarding population and intervention. We did not apply comparison and outcome criteria to web-based tools because we did not expect these to report evaluation studies.

We reported this review according to PRISMA (Preferred Reporting Items for Systematic Reviews and Meta-Analyses) guidelines (see the PRISMA checklist in Multimedia Appendix 2) [39]. We registered our protocol on the Open Science Framework [40].

### Expert Consultations

After extracting information from peer-reviewed papers and tools identified via a Google search, we contacted experts in the field of digital gamified tools (eg, developers and researchers working on the topic in Canada and worldwide who were already known to the research team) to complement our online searches and ensure completeness. Specifically, we sent emails to 12 experts about the results of our searches and asked them to alert us to any games or papers we might have missed.

### Data Charting

We developed a form in Microsoft Excel to guide the charting of data. We pretested and reviewed the form with team members to ensure we were accurately and adequately capturing relevant data. Data charting occurred independently with verification. Specifically, a reviewer (author HH) identified and screened all studies and digital gamified tools for their eligibility. Screening results were verified by a second reviewer (author DG). The data charting was then performed by a reviewer (HH) and again verified by a second reviewer (DG). Any conflicts throughout screening or data charting were resolved by a third reviewer (author ED). From the included papers, we charted data about (1) the type and design of study (developmental or evaluation study, user testing, randomized controlled trial, etc), (2) the vaccine(s) addressed (COVID-19, HPV, etc), (3) the purpose of the study or intervention, (4) the digital gamified tool platform (web based, native mobile app, mobile-enabled web app, virtual reality), and (5) the characteristics of study participants. For the evaluated interventions, we charted data about preselected outcomes that are widely used to predict health-related behaviors and to assess outcomes in studies of interventions about vaccination and immunization [11-14]. Specifically, we extracted data about the tools’ effects on knowledge, attitudes, beliefs (perceived benefits, perceived risks), and behavioral intentions. Emotional, cultural, and social factors can also influence a decision about vaccination [29,30]. Therefore, we also extracted data about other outcomes that the studies may have evaluated. Because we sought to understand all possible effects, we did not prespecify any of these as a primary outcome. We organized the extracted data in tables and synthesized them descriptively.

### Quality Assessment

To assess the quality of the studies that evaluated their interventions, we used the Mixed Methods Appraisal Tool (MMAT) developed by Pluye et al [41]. Two reviewers independently conducted the quality assessment, resolving disagreements through discussion until reaching a consensus. A third and a fourth reviewer (authors HH and HW) intervened to settle any remaining conflicts.

### Data Synthesis

We summarized data using a narrative approach involving framework and content analysis. We classified each digital gamified tool platform using the 4 types of digital gamified tools: web-based tool, native mobile app, mobile-enabled web app, virtual reality tool. For the type of digital gamified tool, we classified web-based tools that explicitly noted their suitability for mobile use (eg, by smartphone or tablet) as mobile-enabled apps. We classified web-based tools without such an explicit statement as web based only, even though they may be functional on mobile devices. For the type and design of study, we grouped randomized designs together, including traditional randomized controlled trials with only 2 study arms and factorial designs with more than 2 study arms. Although these methods are not exactly the same, they all use randomization to minimize potential biases and are therefore functionally equivalent for our purposes of understanding what kinds of evaluations have been undertaken [42]. We summarized the main characteristics of tools, including PICO elements, in a tabular display. We used the PRISMA 2020 flowchart to describe the process of study selection [43].

## Results

### Papers Identified and Scope of Literature

**Figure 1:**
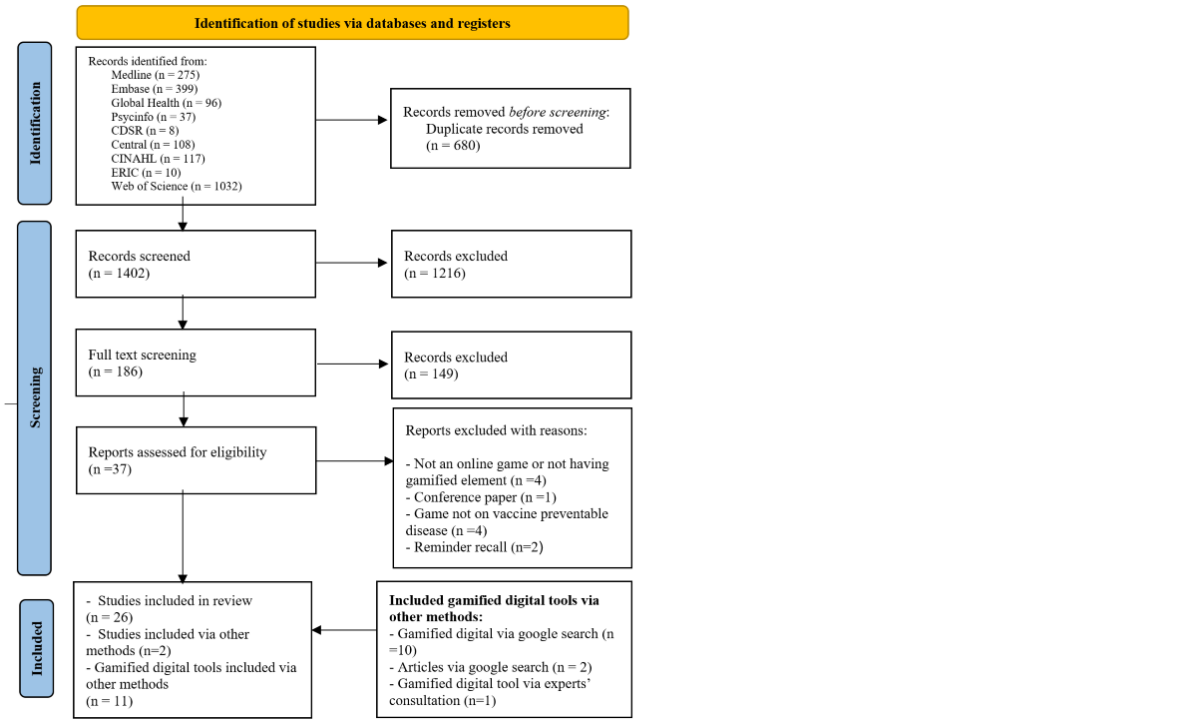
PRISMA flow diagram. PRISMA: Preferred Reporting Items for Systematic Reviews and Meta-Analyses.

We identified a total of 2082 records through database searches. After removing duplicates, we screened 1402 (67.3%) database records. Through Google searches, we identified 10 digital gamified tools and 2 papers. In a private browsing mode search, there was no change in search results. Of the 12 experts contacted, 2 (17%) responded and suggested 2 papers and 2 links, of which 1 (50%) digital gamified tool met the inclusion criteria and was included in our review. Through these methods, our final data set included 28 (2%) peer-reviewed papers and 11 digital gamified tools. Figure 1 shows our PRISMA diagram.

Of the 28 peer-reviewed papers, 7 (25%) explained the development of the tool, 16 (57%) described evaluation, and 2 (7%) reported both development and evaluation of the tool (Table 2). To report our results, we grouped studies together that reported the same tool, meaning 28 peer-reviewed papers reporting on 25 different tools. Of these 25 digital gamified tools, 11 (44%) were web-based tools, 7 (28%) mobile (native mobile or mobile-enabled web) apps, 6 (24%) virtual reality tools, and 1 (4%) offered in both mobile and web-based versions (for details, see Table 2). The most common single vaccines addressed in the tools were influenza (n=6, 24%, tools) and HPV (n=6, 24%, tools). Other tools addressed COVID-19 (n=2, 8%); measles, mumps, influenza, and smallpox (n=2, 8%); a hypothetical disease (n=2, 8%); other vaccine-preventable diseases (n=6, 24%); and the role vaccines play in preventing the spread of disease with no particular vaccine specified (n=1, 4%). Of the 10 digital gamified tools identified via a Google search and 1 suggested by the expert (a total of 11 digital gamified tools; see Table 3), the largest group (n=5, 45%) addressed COVID-19, and the rest were about other vaccine-preventable diseases. The 11 gamified elements identified in the Google search and expert feedback identified 6 types of gamified elements: reward points, serious games, physical trading cards, certificates, role-playing, and quizzes (see Table 3). The most common type was reward points, which appeared in 5 (45%) cases. Two cases used serious games, one case used physical trading cards and reward points, one case used certificates, one case used role-playing, and one case used quizzes. Additional characteristics of the studies included (eg, country of origin, sample size, participant characteristics) are detailed in Multimedia Appendix 3 [31,44-70]. The expanded versions of Table 2 [31,44-70] and Table 3 [71-81] are provided in Multimedia Appendix 4.

**Table 2:** General information about the studies.

Type of study and author(s)			Type of digital gamified tool platform		Type of disease/vaccine	Type and design of study (development or evaluation, iterative design, randomized controlled trial, etc)
**Evaluation studies**						
	Betsch and Böhm [44]	Web-based tool		Hypothetical		Evaluation: online experiment
	Carolan et al [45]	Web-based tool		Measles, mumps, influenza, and smallpox		Evaluation: pre-post study
	Cates et al [31]	Web-based tool		HPV^a^		Evaluation: pilot randomized controlled trial
	Dale et al [46]	Native mobile app		Influenza		Evaluation: nonrandomized trial
	Darville et al [47]	Web-based tool		HPV		Evaluation: randomized controlled trial
	Eley et al [48], McNulty et al [49]	Web-based tool		Bacteria, vaccine-preventable disease		Evaluation: quantitative followed by qualitative research design
	Fadda et al [50], Fadda et al [51]	Native mobile app		MMR vaccines		Evaluation: mixed methods research design
	Ibuka et al [52]	Web-based tool		Hypothetical disease		Evaluation: experimental design
	Kaufman and Flanagan [53]	Web-based tool		Not reported		Evaluation: experimental design
	Lee et al [54]	Native mobile app		Influenza		Evaluation: randomized controlled trial
	Mitchell et al [55], Laplana [56]	Web-based tool		Influenza		Evaluation: pre-post study
	Mottelson et al [57]	Virtual reality tool		COVID-19		Evaluation: randomized controlled trial (2×2 factorial design)
	Nowak et al [58]	Virtual reality tool		Influenza		Evaluation: one-way between-subjects design with random assignment
	Real et al [59]	Virtual reality tool		Influenza		Evaluation: quasi- randomized controlled trial^b^
	Woodall et al [60]	Mobile-enabled web app		HPV		Evaluation: clinic-cluster randomized trial
	Vandeweerdt et al [61]	Virtual reality tool		COVID-19		Evaluation: randomized controlled trial
**Development studies**						
	Amresh et al [62]	Web-based tool		HPV		Development: iterative design
	Bertozzi et al [63] (data extracted for the game related to vaccines)	Web-based tool		Influenza		Development: iterative design
	Carolan et al [64]	Web-based tool		Measles, mumps, influenza, and smallpox		Development: iterative design
	de Araujo Lima et al [66]	Native mobile app		Vaccine-preventable diseases		Development: heuristic evaluation by users, content evaluation by experts
	Kafai et al [65]	Virtual reality		Dragon swooping cough virus to reflect real-life features of infectious viruses, such as Ebola.		Development: user feedback via surveys (asking users questions) and log files (observing user behaviors)
	Real et al [67]	Native mobile app		HPV		Development: usability testing
	Streuli et al [68]	Virtual reality		Pediatric vaccines		Development: Community-based participatory research and co-design
**Development and evaluation studies**						
	Davies et al [69]	Mobile or web app (multiple formats available)		Hepatitis B		Development and evaluation: Participatory Action Research
	Ruiz-López et al [70]	Native mobile app		HPV		Development and evaluation: Iterative design and evaluation via questionnaire

^a^HPV: human papillomavirus.

^b^Allocation to a study arm was performed according to work schedules, which are often arbitrary. We therefore considered this quasi-randomization.

**Table 3:** Tools from Google search and expert suggestions.

Digital gamified tool	Type of disease/vaccine	Type of digital gamified tool platform	Gamification elements (eg, rewards, role-playing, leaderboard, serious game)
Antidote COVID-19 [71]	COVID-19	Native mobile app	Reward points
The Vaccination Game [72]	H11N7 and influenza	Web-based tool	Serious game
Help take down COVID-zilla! [73]	COVID-19	Web-based tool	Role-playing
Just the Vax! [74]	Vaccine-preventable disease	Web-based tool	Reward points
COVID Invaders [75]	COVID-19	Web-based tool	Reward points
Vax Pack Hero [76]	Vaccine-preventable disease	Web-based tool	Reward points and physical trading cards
Flu's Clues [77]	Influenza	Web-based tool	Certificate of completion for solving the influenza mystery
Virus Fighter [78]	COVID-19, influenza, Ebola, measles	Web-based tool	Serious game
Immunization411: for preteens and teens’ online training [79]	Tdap meningococcal vaccine, varicella, HPV^a^, influenza	Web-based tool	Reward points
COVID Chronicles [80]	COVID-19	Web-based tool	Reward points
I Boost^b^ [81]	Vaccine-preventable disease	Web-based tool	Quiz

^a^HPV: human papillomavirus.

^b^Suggested by an expert.

The studies were conducted in 26 different countries, with the majority of studies coming from the United States (n=13, 46%, studies) and the United Kingdom (n=5, 18%, studies). Study populations included students at various levels (elementary school to college, specialty programs, eg, nursing and pediatric residency), parents of vaccine-eligible children, adults from the general population, members of particular sociocultural communities (eg, immigrants, Indigenous peoples), and convenience samples, such as players of a game, attendees of a conference, and employees of an organization. Sample sizes ranged from 8 to 50,286. Whenever papers reported study participant characteristics such as age, sex, gender, ethnocultural identity, or socioeconomic levels, we extracted summary data, as shown in Multimedia Appendix 3.

### Reported Effects of Evaluated Interventions

In total, 18 (64%) of 28 studies evaluated at least 1 of our outcomes of interest, while 11 (39%) studies reported the effects of the evaluated interventions on more than 1 outcome of interest. Summarized outcomes and their MMAT quality assessments are shown in Table 4. Multimedia Appendix 5 provides full details.

**Table 4:** Outcomes of evaluation studies included.

Type of digital gamified tool platform and study			Knowledge (comprehension/understanding, etc)		Attitudes (for/against vaccination, etc)		Beliefs (risk perceptions, etc)		Behavioral intentions (getting vaccinated or not, etc)		Others (eg, emotions)		MMAT^a^ quality score
**Web-based tool**													
	Betsch and Böhm [44]	—^b^		Negative vaccine attitudes with compulsory vaccination		—		Decreased vaccine uptake with compulsory vaccination		Increased level of anger with compulsory vaccination		60% quality criteria met	
	Carolan et al [45]	—		No significant effect on attitudes towards vaccination		—		—		Increased confidence in information needs		80% quality criteria met	
	Cates et al [31]	Increase in knowledge about immunization		—		—		Positive increase in intentions to vaccinate		Increase in vaccination self-efficacy, decisional balance towards vaccination		100% quality criteria met	
	Darville et al [47]	—		—		Positive effects on beliefs towards vaccination		Increase in intentions to vaccinate		—		60% quality criteria met	
	Eley et al [48], McNulty et al [49]	Improvements in knowledge about immunization		—		—		—		—		100% quality criteria met	
	Ibuka et al [52]	—		—		—		—		Free riding in vaccination decisions decreases vaccine acceptance		80% quality criteria met	
	Kaufman and Flanagan [53]	The digital version of the game was less effective at facilitating learning		The digital version of the game was less effective at attitude change		—		—		The digital version of the game was perceived to be complicated to use		20% quality criteria met	
	Mitchell et al [55], Laplana [56]	Increase in knowledge		Positive increase in attitudes for vaccination		—		Increase in vaccine uptake after accessing the game		—		80% quality criteria met (Mitchell et al [55])	
**Mobile app**													
	Dale et al [46]	—		—		—		Positive increase in intentions to vaccinate		—		80% quality criteria met	
	Fadda et al [50], Fadda et al [51]	Improvements in knowledge about immunization		—		—		Increase in intentions to vaccinate		Increase in psychological empowerment and confidence in the decision		80% quality criteria met (Fadda et al [50], Fadda et al [51])	
	Lee et al [54]	—		—		—		Increase in intentions to vaccinate		—		80% quality criteria met	
	Woodall et al [60]	—		—		Increase in beliefs towards vaccination		Increase in intentions to vaccinate		Increase in vaccine confidence		40% quality criteria met	
	Ruiz-López et al [70]	Increase in knowledge after playing the game		—		—		—		—		100% quality criteria met	
**Virtual reality tool**													
	Mottelson et al [57]	—		—		—		Increase in vaccination intention when both the personal and collective benefit of COVID-19 vaccination was communicated		Increase in COVID-19 empathy, vaccination recommendation, and vaccination readiness		80% quality criteria met	
	Nowak et al [58]	—		—		Positive effects on beliefs towards vaccination		Increase in intentions to vaccinate		—		100% quality criteria met	
	Real et al [59]	—		Increase in attitudes in favour of vaccination		—		—		—		60% quality criteria met	
	Vandeweerdt et al [61]	—		—		—		Increase in intentions to vaccinate		Virtual reality intervention increases a sense of collective responsibility		100% quality criteria met	
**Mobile or web app (multiple formats available)**													
	Davies et al [69]	Increase in knowledge about immunization		—		—		—		—		80% quality criteria met	

^a^MMAT: Mixed Methods Appraisal Tool.

^b^Not reported.

#### Effects on Knowledge (Includes Comprehension/Understanding, etc)

Overall, the 28 included studies suggested that digital gamified tools may positively influence knowledge. Of 7 (25%) studies that assessed knowledge, 6 (86%) showed an increase in knowledge about immunization in general [31,48,51,55,69,70]. All these 6 (86%) studies were of high quality (≥80%). One study of low quality (≤25%) reported that a digital game is less effective at increasing knowledge compared to its original board game format [53]. When considering only the high-quality (≥80%) studies, we observed that digital gamified tools are associated with increased knowledge.

#### Effects on Attitudes (for or Against Vaccination)

Overall, digital gamified tools appeared to have mixed effects on attitudes toward vaccination. Of 5 (18%) of 28 studies that assessed attitudes, 2 (40%), one of high quality (≥80%) and the other of medium quality (60%), showed an increase in positive attitudes toward vaccination [55,59]. In addition, 2 (40%) studies, one of high quality (≥80%) and the other of low quality (20%), reported no or less effect on attitudes toward vaccination [45,53], and 1 (20%) study comparing voluntary and compulsory vaccines in a game context showed negative attitudes regarding compulsory vaccination [44]. When considering only the high-quality (≥80%) studies, we observed inconsistent effects on attitudes.

#### Effects on Beliefs (Perceived Benefits, Perceived Risks)

Overall, digital gamified tools demonstrated positive effects on beliefs toward vaccination. In total, 3 (11%) of 28 studies, 1 (33%) of high quality (100%) and 2 (67%) of medium quality (60% and 40%), evaluated the effects of digital gamified tools on beliefs toward vaccination. All 3 (100%) studies showed positive effects on beliefs toward vaccination [47,58,60]. When considering only the high-quality (≥80%) studies, we observed that digital gamified tools are associated with positive beliefs about vaccines.

#### Effects on Behavioral Intentions

Overall, the 28 included studies suggested that digital gamified tools may positively influence intentions to receive vaccines. In total, 11 (39%) studies evaluated the effects of digital gamified tools on behavioral intentions with regard to vaccines. Of these 11 studies, 1 (9%) of medium quality (60%) showed a decrease in vaccination intention when compulsory vaccination was introduced within a game context [44], whereas 10 (91%) studies, 3 (30%) of medium quality (60% and 40%) and 7 (70%) of high quality (≥75%), showed increased intentions to vaccinate [31,46,47,51,54,55,57,58,60,61]. When considering only the high-quality (≥80%) studies, digital gamified tools appeared to be consistently associated with increased vaccination intention.

#### Other Outcomes

In total, 9 (32%) of 28 studies have also evaluated the effects of digital gamified tools on other outcomes. Of these, 4 (44%) studies reported an increase in confidence in vaccines (medium quality=40%) [60], confidence in information needs (high quality=80%) [45], decisional balance in support of vaccination (high quality=100%) [31], and confidence in vaccine decisions (high quality=80%) [50]. In addition, 1 (11%) study of high quality (80%) reported an increase in empathy toward those vulnerable to COVID-19 and vaccination recommendations [57], and 2 (22%) studies of high quality (100% and 80%) reported an increase in vaccination self-efficacy and readiness [31,57]. An increase in psychological empowerment (high quality=80%) [51] and in emotions such as anger toward compulsory vaccination (medium quality=60%) [44] was also reported by 2 (22%) studies. One study of high quality (80%) reported that the concept of free riding decreases vaccine acceptance [52], whereas another study of high quality (100%) reported that virtual reality intervention increases collective responsibility [61]. When considering only the high-quality (≥80%) studies, we observed a variety of positive effects associated with digital gamified tools, including confidence in vaccines, confidence in decisions about vaccines, empathy toward vulnerable people, collective responsibility, psychological empowerment, and vaccination self-efficacy and readiness.

#### Effects of the Platform (Web Based, Mobile, Virtual Reality)

The study designs of the 28 included papers did not permit us to formally compare the effects of different platforms in a robust way. Upon inspection, there did not appear to be a strong effect of the platform. In other words, we did not observe evidence in favor of web-based, mobile, or virtual reality apps over the other 2 types of apps.

## Discussion

### Principal Findings

The broad objective of this scoping review was to map the state of the science regarding digital gamified tools and their effects. In other words, we wished to answer a common question at the intersection of public health and digital health: does gamification encourage vaccination and influence knowledge, attitudes, beliefs, and behaviors related to vaccination? By mapping both published literature and tools currently available online, we observed 2 principal findings.

First, our results suggest that gamification can increase predictors of vaccine uptake, such as knowledge, attitudes, beliefs, behaviors, and vaccination intention. This finding is similar to the findings of a previous review by Montagni et al [82] suggesting that gamification can contribute to changed behaviors and improved knowledge of vaccination. Similarly, other reviews have suggested the potential benefits of gamification for non–vaccination-related behavior change, such as a systematic review suggesting that gamification interventions could be a feasible way to improve health-related outcomes among cancer survivors [83] and another review suggesting their effectiveness in improving physical activity [84]. Such previous work became even more relevant during the COVID-19 pandemic, as many jurisdictions sought to optimize vaccine uptake in the context of an “infodemic” (ie, overabundance of information, true, false, and misleading, about the pandemic and recommended preventive behaviors) [85]. Half of the digital gamified tools identified in our web search addressed COVID-19, suggesting an active interest in using a gamified approach in the pandemic context. Recent research by Plechatá et al [86] published after our data extraction steps were complete suggested that explaining the concept of herd immunity with gamification has a positive impact on the COVID-19 vaccination intention.

Second, our review suggests that although gamification has the potential to enhance the impact of education strategies, gamified tools alone may not wholly address gaps in vaccine acceptance and uptake. Although some of the identified tools did increase vaccination, the increases did not fully close gaps between previous and desired vaccine uptake. This finding aligns with those of Tozzi et al [87], which suggested that promising results could be achieved by combining gamification with educative and informative tools to improve immunization programs. This finding also aligns with previous reviews suggesting the use of digital gamified interventions as a public health tool of interest in enhancing vaccine uptake [82,88]. Further research published by Real et al [89] after our systematic search similarly observed that integrating gamification, such as virtual reality, in training modules enhances uptake of the HPV vaccine. Integrating gamified features may work because they make digital tools acceptable and more fun to use and may reduce the chances of people feeling pushed toward vaccination. In parallel, gamification may be a promising strategy for increasing knowledge, skills, and confidence among health professionals engaging in discussions about vaccines with their patients [90,91].

In addition to these findings drawn directly from our review of the included tools, we offer a broader observation based on the contents of this scoping review, along with the larger landscape of vaccine acceptance research: context is key. Although an engaging approach may work for some groups or in some situations, it may be less well accepted among other groups and in other situations. For instance, a casual and approachable style of communication will work for the younger audience to convey vaccine information but might be deemed insufficient to health care professionals in a more formal setting, such as hospitals. A good understanding of the factors associated with low vaccine acceptance at the local level is needed prior to developing gamified tools [92]. Future research in this area should consider possible contextual factors, such as local culture, social and demographic characteristics of users, and different influences on vaccine hesitancy and acceptance in different regions. To help better match games to the context(s) in which they will be played, when developing games, developers and researchers may wish to consider involving potential players from different contexts early and often. This aligns with previous work [93,94] suggesting that involving users earlier in developing tools may help in designing interventions suitable for a targeted context. One of the examples in our review was an intervention by Cates et al [31] designed to explain HPV vaccines to teenagers using a “secret garden” theme. Involving potential game players early in the development of the game may have contributed toward its positive effects on vaccination intention.

The implications of this research extend beyond the immediate reported effects of gamified tools and delve into the strategic dimensions of public health policy and communication efforts. Considering the insights gleaned from the findings, this study supports a comprehensive and well-informed approach to integrating gamification into strategies for promoting vaccination. As gamification continues to demonstrate its potential in enhancing vaccine uptake, it is crucial to navigate this terrain thoughtfully, considering the various factors that influence its impact. This includes not only the technological and behavioral aspects but also the larger sociocultural context in which vaccination decisions are made. Therefore, our study emphasizes the importance of a comprehensive approach that fosters a mutually beneficial relationship between technological innovation, evidence-based strategies, and an intricate understanding of local contexts. This approach has the potential to make gamification a sustainable and adaptable tool in the arsenal of public health interventions, rather than just a passing trend.

The review does not find a clear advantage for any platform in terms of reported effects. It was challenging to measure the impact of the platforms on behavioral outcomes and calls for more focused research to better understand the specific elements within each platform that drive behavior change. In essence, our study suggests that the reported effects of an app may not be solely determined by its platform but rather by the strategic incorporation of mechanics and elements that facilitate the desired behavior change.

Gamification can influence knowledge, attitudes, and beliefs about vaccines, which can affect vaccine uptake. This is consistent with theories of change proposing that cognitive changes can lead to behavioral outcomes. Although our study mainly examines the immediate effects of gamification on these cognitive aspects, it also offers some implications for using gamification as a potentially viable strategy to improve vaccine acceptance.

### Strengths and Limitations

Our study has 5 main limitations. First, because we aimed to capture all relevant evidence and examples, as is typical in a scoping review, we included a broad range of study designs and did not draw conclusions about the relative advantages or disadvantages of different game platforms and features. Given the rapid growth within this field of research, it would be difficult to truly prioritize evidence according to quality criteria at this point. In the future, it may be possible to conduct a systematic review and meta-analysis, restricting included studies to randomized experiments or randomized controlled trials. Such future work may include approaches such as a network meta-analysis to allow for comparison of the effects of different game types or game features. Based on the existing literature, it is difficult to conclude whether certain games are more or less likely to achieve their aims. Second, our results may be influenced by publication bias. It is possible that groups that have developed digital gamified tools that showed disappointing results simply did not publish their studies. This bias could lead to an overestimation of the reported effects of these tools. This highlights the importance of further research to fully understand the real impact of these tools and thus accurately inform policy decisions about the development and use of these tools. Third, and related to the previous 2 points, the rapid growth in this area may mean that we missed more recent evidence in literature published after January 2022 and web searches after May 2022. Fourth, the majority of digital gamified tools on vaccination represented in publications and online were developed in high-income countries. This finding aligns with the findings of previous work by Ohannessian et al [88], who also reported a predominance of high-income countries. This may reflect more widespread internet access and resources for developing digital gamified tools in high-income countries. It may also reflect publication bias in the scientific literature (ie, there may be fewer papers written about digital gamified tools in lower-income countries) and online (ie, tools developed and published in lower-income countries may not be ranked highly by search engines and therefore may not have appeared in our web searches). Tools developed in lower-income countries may also take different forms; for example, they may be text message–based interventions (with or without gamification) rather than web-based tools and therefore would be less likely to be identified in web searches. Analog games from high-income countries were similarly excluded from the scope of our study [95]. Nondigital games, such as board and card games, have demonstrated positive impacts on educational knowledge, cognitive function, and social interactions [96,97]. Such games can support diverse learning across subjects and settings, fostering interactions that develop skills, such as computational thinking and teamwork, and have positive impacts on academic achievement and vocabulary acquisition compared to digital games [97-99]. We restricted our scoping review to digital gamified tools because the review was intended to provide an evidence base for digital game development. Although nondigital games are also potentially useful interventions, the implementation and distribution of such interventions is more challenging, especially in a geographically dispersed country, such as Canada. Fifth, and finally, as we used Google and private browsing in Google, there may be a possibility that different search engines would provide different results.

This study also has 2 main strengths. First, by systematically examining the current literature and currently available tools online, we were able to offer an updated overview of the potential effects of including gamification in digital tools about vaccination. Second, by conducting a scoping review to broadly map the literature, future work can more easily identify and select key outcomes for systematic reviews and meta-analyses in this domain.

### Conclusion

Digital gamified tools have the potential to improve vaccine uptake by increasing knowledge and promoting positive attitudes, beliefs, behaviors, and vaccination intention. Further evaluations of these innovative digital tools, including head-to-head comparisons of different features and different platforms, will add more knowledge about what works and what does not in order to achieve public health goals more efficiently. In the wider context of health policy, digital gamified tools may be useful components of multifaceted strategies to improve vaccination rates throughout society.

## Appendix

Multimedia Appendix 1 Search strategy.

Multimedia Appendix 2 PRISMA-ScR checklist.

Multimedia Appendix 3 Characteristics of the studies included in the review.

Multimedia Appendix 4 Expanded version of Table 2 (general information about the studies) and Table 3 (tools from Google search and expert suggestions).

Multimedia Appendix 5 MMAT quality assessment. MMAT: Mixed Methods Appraisal Tool.

## Abbreviations

HPV: human papillomavirus
MMAT: Mixed Methods Appraisal Tool
PICO: Population, Intervention, Comparison, and Outcome
PRISMA: Preferred Reporting Items for Systematic Reviews and Meta-Analyses
