# Supplementary material for: Digital Gamification Tools to Enhance Vaccine Uptake: Scoping Review
Source: JMIR Serious Games. 2024 Feb 29;12:e47257. doi: 10.2196/47257 (PMC10906656; doi:10.2196/47257)
Supplement: Multimedia Appendix 1 [file games_v12i1e47257_app1.docx]

## **Multimedia Appendix 1:**

### **Search strategy**

#### **Search strategy for Ovid (Medline)**

Search date: 2022-01-26

| # | Search strategy: | **Number of results** |
| --- | --- | --- |
| 1 | exp Vaccination/ OR Vaccination Refusal/ OR Anti-Vaccination Movement/ OR exp vaccines/ OR immunization programs/ OR  (vaccin* OR immuni#ation* OR immuni#e* OR immuni#ing OR anti vax* OR antivaccin* OR antivax*).ti,ab |  |
| 2 | Video Games/ OR Virtual Reality/ OR Augmented Reality/ OR  (game? OR gamif* OR gaming OR videogame* OR ((mobile* OR smartphone* OR phone* OR web) ADJ3 (app? OR application*))).ti. OR  (((electronic* OR computer* OR mobile* OR smartphone* OR phone* OR interactive* OR video* OR digital* OR serious* OR online* OR on line* OR therap* OR app? OR application* OR web) ADJ6 game?) OR gamif* OR gaming OR videogame*).ab OR  ((virtual* OR augment*) ADJ realit*).ti,ab |  |
| 3 | 1 AND 2 | 275 |

#### **Search strategy for Ovid (Embase)**

Search date: 2022-01-26

| # | Search strategy: | **Number of results** |
| --- | --- | --- |
| 1 | exp vaccination/ OR vaccination coverage/ OR vaccination refusal/ OR anti-vaccination movement/ OR exp vaccine/ OR  (vaccin* OR immuni#ation* OR immuni#e* OR immuni#ing OR anti vax* OR antivaccin* OR antivax*).ti,ab |  |
| 2 | exp video game/ OR virtual reality/ OR augmented reality/ OR  (game? OR gamif* OR gaming OR videogame* OR ((mobile* OR smartphone* OR phone* OR web) ADJ3 (app? OR application*))).ti. OR  (((electronic* OR computer* OR mobile* OR smartphone* OR phone* OR interactive* OR video* OR digital* OR serious* OR online* OR on line* OR therap* OR app? OR application* OR web) ADJ6 game?) OR gamif* OR gaming OR videogame*).ab OR  ((virtual* OR augment*) ADJ realit*).ti,ab |  |
| 3 | 1 AND 2 | 399 |

#### **Search strategy for Ovid (Global Health)**

Search date: 2022-01-26

| # | Search strategy: | **Number of results** |
| --- | --- | --- |
| 1 | exp vaccination/ OR exp vaccines/ OR immunization programmes/ OR  (vaccin* OR immuni#ation* OR immuni#e* OR immuni#ing OR anti vax* OR antivaccin OR antivax*).ti,ab |  |
| 2 | video games/ OR computer games/ OR  (game? OR gamif* OR gaming OR videogame* OR ((mobile* OR smartphone* OR phone* OR web) ADJ3 (app? OR application*))).ti. OR  (((electronic* OR computer* OR mobile* OR smartphone* OR phone* OR interactive* OR video* OR digital* OR serious* OR online* OR on line* OR therap* OR app? OR application* OR web) ADJ6 game?) OR gamif* OR gaming OR videogame*).ab OR  ((virtual* OR augment*) ADJ realit*).ti,ab |  |
| 3 | 1 AND 2 | 96 |

#### **Search strategy for Ovid (Psycinfo)**

Search date: 2022-01-26

| # | Search strategy: | **Number of results** |
| --- | --- | --- |
| 1 | Immunization/ OR  (vaccin* OR immuni#ation* OR immuni#e* OR immuni#ing OR anti vax* OR antivaccin OR antivax*).ti,ab |  |
| 2 | Computer Games/ OR Digital Game-Based Learning/ OR Digital Gaming/ OR Avatars/ OR exp Virtual Reality/ OR  (game? OR gamif* OR gaming OR videogame* OR ((mobile* OR smartphone* OR phone* OR web) ADJ3 (app? OR application*))).ti. OR  (((electronic* OR computer* OR mobile* OR smartphone* OR phone* OR interactive* OR video* OR digital* OR serious* OR online* OR on line* OR therap* OR app? OR application* OR web) ADJ6 game?) OR gamif* OR gaming OR videogame*).ab OR  ((virtual* OR augment*) ADJ realit*).ti,ab |  |
| 3 | 1 AND 2 | 37 |

#### **Search strategy for Ovid (Cochrane Database of Systematic Reviews)**

Search date: 2022-01-26

| # | Search strategy: | **Number of results** |
| --- | --- | --- |
| 1 | Vaccination.kw OR Vaccination Refusal.kw OR Anti-Vaccination Movement.kw OR vaccines.kw OR immunization programs.kw OR  (vaccin* OR immuni#ation* OR immuni#e* OR immuni#ing OR anti vax* OR antivaccin* OR antivax*).ti,ab,kw |  |
| 2 | Video Games.kw OR Virtual Reality.kw OR Augmented Reality.kw OR  (game? OR gamif* OR gaming OR videogame* OR ((mobile* OR smartphone* OR phone* OR web) ADJ3 (app? OR application*))).mp OR  (((electronic* OR computer* OR mobile* OR smartphone* OR phone* OR interactive* OR video* OR digital* OR serious* OR online* OR on line* OR therap* OR app? OR application* OR web) ADJ6 game?) OR gamif* OR gaming OR videogame*).mp OR  ((virtual* OR augment*) ADJ realit*).mp |  |
| 3 | 1 AND 2 | 8 |

#### **Search strategy for Ovid (Cochrane Central Register of Controlled Trials)**

Search date: 2022-01-26

| # | Search strategy: | **Number of results** |
| --- | --- | --- |
| 1 | Vaccination.kw,sh OR Vaccination Refusal.kw,sh OR Anti-Vaccination Movement.kw,sh OR vaccines.kw,sh OR immunization programs.kw,sh OR  (vaccin* OR immuni#ation* OR immuni#e* OR immuni#ing OR anti vax* OR antivaccin* OR antivax*).mp |  |
| 2 | Video Games.kw,sh OR Virtual Reality.kw,sh OR Augmented Reality.kw,sh OR  (game? OR gamif* OR gaming OR videogame* OR ((mobile* OR smartphone* OR phone* OR web) ADJ3 (app? OR application*))).mp OR  (((electronic* OR computer* OR mobile* OR smartphone* OR phone* OR interactive* OR video* OR digital* OR serious* OR online* OR on line* OR therap* OR app? OR application* OR web) ADJ6 game?) OR gamif* OR gaming OR videogame*).mp OR  ((virtual* OR augment*) ADJ realit*).mp |  |
| 3 | 1 AND 2 | 108 |

#### **Search strategy for EBSCO (CINAHL Complete)**

Search date: 2022-01-26

| # | Search strategy: | **Number of results** |
| --- | --- | --- |
| S1 | (MH "Immunization+") OR (MH "Attitude to Vaccines") OR (MH "Vaccines+") OR TI (vaccin* OR immuni?ation* OR immuni?e* OR immuni?ing OR "anti vax*" OR antivaccin* OR antivax*) OR AB (vaccin* OR immuni?ation* OR immuni?e* OR immuni?ing OR "anti vax*" OR antivaccin* OR antivax*) |  |
| S2 | (MH "Video Games+") OR (MH "Virtual Reality") OR TI (game# OR gamif* OR gaming OR videogame* OR ((mobile* OR smartphone* OR phone* OR web) N2 (app# OR application*)) OR ((virtual* OR augment*) N0 realit*)) OR AB (((electronic* OR computer* OR mobile* OR smartphone* OR phone* OR interactive* OR video* OR digital* OR serious* OR online* OR "on line*" OR therap* OR app# OR application* OR web) N5 game#) OR gamif* OR gaming OR videogame* OR ((virtual* OR augment*) N0 realit*)) |  |
| S3 | S1 AND S2 | 117 |

#### **Search strategy for EBSCO (ERIC)**

Search date: 2022-01-26

| # | Search strategy: | **Number of results** |
| --- | --- | --- |
| S1 | DE "Immunization Programs" OR  TI (vaccin* OR immuni?ation* OR immuni?e* OR immuni?ing OR "anti vax*" OR antivaccin* OR antivax*) OR AB (vaccin* OR immuni?ation* OR immuni?e* OR immuni?ing OR "anti vax*" OR antivaccin* OR antivax*) |  |
| S2 | DE "Video Games" OR DE "Computer Games" OR DE "Computer Simulation" OR  TI (game# OR gamif* OR gaming OR videogame* OR ((mobile* OR smartphone* OR phone* OR "web") N2 ("app" OR application*)) OR ((virtual* OR augment*) N0 realit*)) OR AB (((electronic* OR computer* OR mobile* OR smartphone* OR phone* OR interactive* OR video* OR digital* OR serious* OR online* OR "on line*" OR therap* OR "app" OR application* OR web) N5 game#) OR gamif* OR gaming OR videogame* OR ((virtual* OR augment*) N0 realit*)) |  |
| S3 | S1 AND S2 | 10 |

#### **Search strategy for Web of Science**

Search date: 2022-01-27

| # | Search strategy: | **Number of results** |
| --- | --- | --- |
| 1 | TS=(vaccin* OR immuni?ation* OR immuni?e* OR immuni?ing OR (anti NEAR/0 vax*) OR antivaccin* OR antivax*) |  |
| 2 | TS=(game$ OR gamif* OR gaming OR videogame* OR ((mobile* OR smartphone* OR phone* OR web) NEAR/3 (app$ OR application*)) OR ((virtual* OR augment*) NEAR/1 realit*)) |  |
| 3 | AB=(((electronic* OR computer* OR mobile* OR smartphone* OR phone* OR interactive* OR video* OR digital* OR serious* OR online* OR (on NEAR/0 line) OR therap* OR app$ OR application* OR web) NEAR/6 game$) OR gamif* OR gaming OR videogame*) |  |
| 4 | 1 AND (2 OR 3) | 1032 |

#### **Search strategy for Google**

**Search strategy: Google** 2022-05-05

1. vaccination|vaccine|immunization "electronic game"|"computer game"|"mobile game"|"interactive game"
2. vaccination|vaccine|immunization "video game"|"digital game"|"serious game"|"online game"|"therapeutic game"
3. vaccination|vaccine|immunization "virtual reality"|"augmented reality"
4. vaccination|vaccine|immunization "electronic|computer|mobile|interactive AROUND(3) game"
5. vaccination|vaccine|immunization "video|digital|serious|online|therapeutic AROUND(3) game"
