# Supplementary material for: Digital Gamification Tools to Enhance Vaccine Uptake: Scoping Review
Source: JMIR Serious Games. 2024 Feb 29;12:e47257. doi: 10.2196/47257 (PMC10906656; doi:10.2196/47257)
Supplement: Multimedia Appendix 3 [file games_v12i1e47257_app3.docx]

# **Multimedia appendix 3: Characteristics of the studies included in the review:**

| **Authors name and references of the studies** | **Country** | **Participant/population** | **Sample size** | **Age** | **Sex/Gender^^[[1]](#footnote-1)^^** | **Other socio-demographic characteristics reported (ethnocultural identity, socio-economic level)** |
| --- | --- | --- | --- | --- | --- | --- |
| Amresh et al., 2019 [62] | United States | Adolescents and their guardians | n = 8 guardian-adolescent pairs (4 guardian-boy pairs and 4 guardian-girl pairs | Guardians: Aged from 31 to 55 years (mean = 40 years)  Adolescents: Aged from 11 to 14 (mean = 12) | Guardians: Female = 6  Male = 3  Adolescents:  Female = 4  Male = 4 | *Ethnocultural identity n (%)*:  Non-Hispanic White: 4 (50%);  Hispanic/Latino: 2 (25%);  Asian:1 (12.5%); Black: 1 (12.5) |
| Bertozzi et al., 2013 [63]] (Data extracted for the game related to vaccines) | United States | School-age children | n = 12 | Not reported | Not reported | Not reported |
| Betsch & Böhm, 2016 [44] | Germany | University students | n = 297 | Mean (SD)= 23.11 years (3.86) | Female = 59.9% | Not reported |
| Carolan et al., 2018 [45] | United Kingdom | Students, trainee teachers | n = 92 | Students: all aged between 16 and 18 years  Other groups age not reported | Not reported | Not reported |
| Carolan et al., 2018 [64] | United Kingdom | School students | n= 63 | Aged 14 and 15 years | Female n(%) = 29 (46.03%)  Male n (%) = 34 (53.97%) | *Ethnocultural identity n (%)*:  White British: 59 (93.65%);  Asian/Asian British: 3 (5.45%); Mixed Ethnic Background: 1 (1.59%) |
| Cates et al., 2020 [31] | United States | Preteens and parents recruited at clinical sites | Preteens:  n = 28 in intervention group, n = 27 in comparison group  Parents:  Wave 1, n = 10 Wave 2 and 3, n = 20 | Preteens: Aged 11 to 12 years  Parents: Not reported | Preteens: n(%)  Female = 16 (57%) in intervention group, 14 (52%) in comparison group  Parents: Not reported | *Ethnocultural identity n (%)*:  Preteens:  71% (20/28) white intervention group, and 82% (22/27) were white comparison group.  Parents: Not reported |
| Dale et al., 2019 [46] | Canada | Registered users (Carrot Rewards application) | n = 30,538 | Mean = 35.96 years (SD = ±12.94) | Female n(%) = 19,916 (65.2%) | *Ethnocultural identity n (%)*:  *Socio-economic:*  Less than $20,000: 940 (9.2%)  $20,000–40,000: 1,557 (15.2%)  $40,000–60,000: 1,665 (16.3%)  $60,000–80,000: 1,345 (13.1%)  $80,000–100,000: 1,200 (11.7%)  $100,000–150,000: 1,398 (13.6%)  Over $150,000: 692 (6.8%) Don’t know/rather not say: 1,432 (14.0%) |
| Darville et al., 2018 [47] | United States | College men (non-recipient of the HPV vaccine) | n= 108  (condition 1: n = 27 (25%); condition 2: n= 24 (22.2%); condition 3: n = 29 (26.9%); condition 4: n: 28 (25.9%) | Aged 18 to 20 years n(%) = 39 (36.1%);  Aged 21 to 23 year n(%) = 45 (41.7%)  Aged 24 to 26 n(%) = 24 (22.2%) | Male n(%) = 108 (100%) | *Ethnocultural identity n (%)*:  American Indian or Alaskan Native n= 0, 0%;  Asian n=35, 32.4%; Black or African American n= 0, 0%; Hispanic n= 6,5.6%; Native Hawaiian or Pacific Islander n= 16, 14.8%; White or Caucasian n=46, 42.6%; Bi-racial or Multi-racial n= 5, 4.6%  Not reported |
| Davies et al., 2015 [69] | Australia | Community and conference delegates | Community launch group: n = 16  Conference delegate group: n=56 | Mean age:  Community launch group = 34 years  Conference delegate group: 45 years | Female n(%)=  Community launch group = 12 (75%)  Conference delegate group n(%) = 50 (75%) | *Ethnocultural identity n(%)*:  *Indigenous status*:  Community launch group = 15 (94%)  Conference delegate group = 12 (21%) |
| Eley et al., 2019 [48]; McNulty et al., 2011 [49] | United Kingdom | Junior and senior students from 5 educational providers | n = 473 (junior students: n =1 23, senior students: n = 350) | All aged between 7 to 16 years | Not reported | Not reported |
| Fadda et al., 2017 [50]; Fadda et al., 2018 [51] | Italy | Parents of young children | n = 184 (experimental group 1 n (%)= 48(26%); experimental group 2 n(%)=45 (24%), experimental group 3 n(%) = 47(26%), experimental group 4 n(%)= 44 (24%) | Mean age = 34.2 years (SD = 4.66)  (Mean age  experimental group 1 = 33.44 (SD = 4.27)  experimental group 2 = 34.49 (SD = 4.46)  experimental group 3 Mage= 33.98, (SD = 4.86)  experimental group 4 = 35 (SD = 5.06) | Overall Women n (%) = 174 (95%)  Overall Men n (%) = 10 (5 %)  *Women:* experimental group 1 = 43 (25%) experimental group 2 = 43 (25%)  experimental group 3 = 46 (26%) experimental group 4 = 42 (24%)  *Men:* experimental group 1 = 5 (50%) experimental group 2 = 2 (20%) experimental group 3 = 1 (10%) experimental group 4 = 2 (20%) | *Ethnocultural identity n(%)*:  Italy = 179 (97%);  Brazil = 1 (1%); Morocco = 1 (1%);  Mexico = 1 (1%) |
| Ibuka et al., 2014 [52] | United States | Undergraduate students | n = 269 (29 groups formed by 8 to 10 participants) | Not reported | Not reported | Not reported |
| Kafai et al., 2017 [65] | United States | Players of an online community | n = 5,835 (recruitment source 1 = 835, recruitment source 2 = 5,000) | Not reported | Not reported | Not reported |
| Kaufman & Flanagan, 2013 [53] | United States | Middle and high school students | n = 26 schools  (no students sample size provided) | Not reported | Not reported | Not reported |
| Lee et al., 2020 [54] | United States | Adults from a large national health plan (Humana) | N=50,286; (incentive arm n = 16,762, generic arm n=16,762; Control arm n=16,762 | Mean age: incentive arm = 41.5 years (SD = 10.9)  generic arm = 4 years (SD = 11.0)  control arm = 1.6 years (SD = 10.9) | Female n(%):  incentive arm = 10,357 (61.9%); generic arm= 10,487 (62.7%);  control arm= 10,400 (62.1%) | Not reported |
| de Araujo Lima et al., 2022 [66] | Brazil | Nursing experts and nursing students | Nursing experts n = 49 Nursing students n = 15 | Mean age =  Nursing experts = 44.04 years)  Nursing students = 22.13 years | Female =  Nursing experts = 43 (88%)  Nursing students = 14 (93%)  Male =  Nursing experts = 6 (12%)  Nursing students = 1 (7%) | Not reported |
| Mitchell et al., 2021[55]; Laplana, 2019 [56] | United Kingdom | Nursing students, from year 1, 2 and 3 | n = 430  Year 1 n = 229  Year 2 n = 105  Year 3 n = 96 | Not reported | Not reported | Not reported |
| Mottelson et al., 2021 [57] | Multiple countries (United States, United Kingdom, Germany, Canada, France, Spain, Poland, Netherlands, Italy, Sweden, Mexico, Denmark, Ireland, Turkey, Argentina, Brazil, Japan) | Adults | n = 282 | Mean age = 28.9 years (SD = 9.7) | Female n(%) = 20 (7.1%);  Male n(%) = 255 (90.4%);  Non-binary n(%) = 7 (2.5%) | Not reported |
| Nowak et al., 2020 [58] | United States | Adults who self-reported not receiving a flu vaccination or ‘‘probably not” or ‘‘definitely not” receive one for the 2017–18 flu season | n = 171   (Immersive virtual reality n = 48  Video n = 41 E-pamphlet n =3  ;No exposure/control n = 43) | 57.9% were aged between 18 to 35 years | 67.3% were female | Not reported |
| Real et al., 2017 [59] | United States | Pediatric residents | n = 45 (intervention n = 24, control n = 21) | Aged between 25 and 29 years =  Intervention n = 188 (75.0%)  Control n = 15 (71.4%)  Aged ≥ 30 yrs =  Intervention n = 6 (25.0%) Control n = 6 (28.6%) | Female =  Intervention n = 16 (66.7%) Control n =  16 (76.2%)  Male = Intervention n = 8 (33.3%) Control n = 5 (23.8%) | Ethnocultural identity:  White =  Intervention =19 (79.2%)  Control= 12 (57.1%)  Nonwhite =  Intervention = 5 (20.8%)  Control = 9 (42.9%) |
| Real et al., 2021 [67] | United States | Pediatric residents | n = 15 | < 30 years n = 10 (66.7%)  ≥ 30 years n = 5 (33.3%) | Female = 12 (80%)  Male = 3 (20%) | *Ethnocultural identit*y:  White = 10 (66.7%)  Non-White = 5 (33.3%) |
| Ruiz-Lópezl et al., 2019 [70] | Norway | Beta testing study: Employees of the Cancer Registry of Norway  Focus Group 1: Norwegian Women’s Public Health Association,  Focus Group 2: high school students living in Oslo | Beta testing study n = 40  Focus group 1 n = 6  Focus group 2 n = 23 | Beta testing study = 30 and 60 years  Focus group 1 = age varied from 40 and 60 years  Focus group 2 = age varied from 16 and 18 years | Beta testing study = male and female  Focus group 1 = all female  Focus group 2 = 10 female and 13 male | Not reported |
| Streuli et al., 2021 [68] | United States (Somali immigrant community) | Somali refugees participated at the offices of a community-based organisation, Somali Family Service, in San Diego, California and expert advisors who specialise in healthcare | n= 67  Somali refugees n = 60  expert advisors n = 7 | Not reported | Majority of participants were female (n not specified) | Socioeconomic statistics of this region include a median household income of $39 330. |
| Woodall et al., 2021 [60] | United States | Parent-adolescent daughter pairs within clinics | n = 82 pairs | Mean age =  Parents = 38.96 years (SD = 9.64)  Daughters = 12.05 years (SD = 1.08) | Female:  Parents = 92.5% | *Ethnocultural identity:*  Hispanic = 38.5%  American Indian/Native Alaskan = 6.2%  Asian = 1.2%  Caucasian = 37.8%  Unspecified = 12.3%  Missing information = 3.7% |
| Vandeweerdt et al., 2022 [61] | Denmark | Adults | n = 222 | Age varied bewteen18 and 63 years | 39% were female | Not reported |

1. Many studies did not specify whether they asked participants about their sex (i.e., the biological sex they were assigned at birth, typically male or female) or gender (i.e., the gender with which they identify, which may be male or men, female or women, non-binary, and other genders.) In this table, we report sex and/or gender statistics together, however they appeared in the original papers. [↑](#footnote-ref-1)
