# Supplementary material for: Digital Gamification Tools to Enhance Vaccine Uptake: Scoping Review
Source: JMIR Serious Games. 2024 Feb 29;12:e47257. doi: 10.2196/47257 (PMC10906656; doi:10.2196/47257)
Supplement: Multimedia Appendix 4 [file games_v12i1e47257_app4.docx]

# **Expanded version of table 2: General information of the studies**

| **Authors name and references of the study** | **Purpose of the study** | **Context of the study: Entertainment, State & Government Healthcare, Education, Scientific Research (i.e., setting where it took place)** | **Type of gamified digital tool platform** | **Type of disease/vaccine** | **Type and design of the study**  **(Development or evaluation, iterative design, randomized controlled trial etc.)** |
| --- | --- | --- | --- | --- | --- |
| **Evaluation studies** | | | | | |
| Betsch & Böhm, 2016 [44] | To assess the effect of partial compulsory vaccination on the uptake of other voluntary vaccines using behavioural vaccination game | Educational setting: University students of various academic disciplines | Web-based | Hypothetical | Evaluation: Online experiment |
| Carolan et al., 2018 [45] | To assess both digital simulation-based and traditional educational interventions on attitudes towards vaccination or level of confidence in knowledge of vaccination | Educational setting: School | Web-based | Measles, mumps, influenza and smallpox | Evaluation: Pre-post study |
| Cates et al., 2020 [31] | To evaluate the acceptability and feasibility of using a serious video game “the Land of Secret Gardens” about Human papillomavirus (HPV) vaccination | Healthcare setting 36 primary care practices | Web-based | Human papillomavirus (HPV) | Evaluation: Pilot Randomized Controlled Trial |
| Dale et al., 2019 [46] | To evaluate the uptake of the Carrot Flu Campaign educational quiz and to determine if mobile ‘‘push” notifications, plus loyalty point incentives, resulted in users visiting a sponsored pharmacy | State and government: The Carrot Flu Campaign was delivered to all valid Carrot users in British Columbia, Canada | Native mobile application | Influenza | Evaluation: Non-randomized trial |
| Darville et al., 2018 [47] | To evaluate the effectiveness of a digital gaming intervention aimed at increasing HPV risk perceptions, self efficacy and behavioral intention | Educational and Scientific Research: Research-intensive university | Web-based | Human papillomavirus (HPV) | Evaluation: Randomized controlled trial |
| Eley et al., 2019 [48]; McNulty et al., 2011 [49] | To determine students’ baseline knowledge, views on the 2 e-Bug games (Body Busters, and Stop the Spread), and knowledge improvement | Educational setting: 5 UK educational provisions | Web-based | Bacteria, vaccine preventable disease | Evaluation: Quantitative followed by qualitative research design |
| Fadda et al., 2017 [50]; Fadda et al., 2018 [51] | To look into the effects of two smartphone-based interventions targeting MMR vaccination knowledge and psychological empowerment | Online study | Native mobile application | MMR vaccines | Evaluation: Mixed-methods research design |
| Ibuka et al., 2014 [52] | To examine an individual’s decision about vaccination in a group setting for a hypothetical disease using a computerized experimental game | Educational setting: Rutgers University | Web-based | Hypothetical disease | Evaluation: Experimental design |
| Kaufman & Flanagan, 2013 [53] | To describe the design of the analog and digital versions of POX and to assess if the analog to digital format influence players’ perceptions of the game and/or impact the effectiveness of the game | Educational setting: Middle and high school students | Web-based | Not reported | Evaluation: Experimental design |
| Lee et al., 2020 [54] | To assess the effect of large-scale messaging combined with individualized incentives on influenza vaccination rates | Online study | Native mobile application | Influenza | Evaluation: Randomized controlled trial |
| Mitchell et al., 2021 [55]; Laplana, 2019 [56] | To evaluate the effect of a serious game about influenza, on nursing student attitude, knowledge and uptake of the influenza vaccination | Educational setting: School of Nursing and Midwifery Queen's University Belfast | Web-based | Influenza | Evaluation: Pre-post study |
| Mottelson et al., 2021 [57] | To investigate the effectiveness of a novel automated intervention strategy for decreasing vaccine hesitancy targeted at a young audience | Online study | Virtual reality | COVID 19 | Evaluation:  Randomized controlled trial (2x2 factorial design) |
| Nowak et al., 2020 [58] | To examine whether supplementing vaccine information statements (VIS) with an immersive virtual reality (VR), short video or electronic pamphlet story designed to convey the community immunity benefits of influenza vaccination would improve perceptions, beliefs, confidence and intentions | Educational and scientific setting: A clinical translational research unit affiliated with the university of Georgia | Virtual reality | Influenza | Evaluation: A one-way between-subjects design with random assignment |
| Real et al., 2017 [59] | To assess the impact of an immersive virtual reality (VR) communication curriculum that focused on vaccine hesitancy on rates of influenza vaccine refusal | Educational and healthcare setting: The Cincinnati Children’s Hospital, pediatric primary care center | Virtual reality | Influenza | Evaluation: Quasi- randomized controlled trial^^[[1]](#footnote-1)^^ |
| Woodall et al., 2021 [60] | To report the results from a small-scale randomized trial testing a mobile web app to encourage HPV vaccination | Clinical setting: Pediatric clinics | Mobile-enabled web application | Human papillomavirus (HPV) | Evaluation: A clinic-cluster randomized trial |
| Vandeweerdt et al., 2022 [61] | To investigate whether vaccination intention is increased by a gamified immersive VR experience showing how community immunity works. | Educational and public setting: Public park and University | Virtual reality | COVID-19 | Evaluation: Randomized controlled trial |
| **Development studies** | | | | | |
| Amresh et al., 2019 [62] | To develop and pilot test a game-based intervention | Clinical setting | Web-based | Human papillomavirus (HPV) | Development: Iterative design |
| Bertozzi et al., 2013 [63] *(Data extracted for the game related to vaccines) | To discuss the creation of two serious games, one developed about childhood vaccines | Healthcare setting: School-aged children of American and immigrant families | Web-based | Influenza | Development: Iterative design |
| Carolan et al., 2018 [64] | To document the development of SimFection as a robust educational tool for its target audience | Educational setting: Undergraduate biology students at the university | Web-based | Measles, mumps, influenza and smallpox | Development: Iterative design |
| Kafai et al., 2017 [65] | To describe the design and impact of a virtual epidemic in a massive online community called Whyville.net | Online study | Virtual reality | Dragon Swooping Cough virus to reflect real-life features of infectious viruses like Ebola. | Development: User feedback via surveys (asking users questions) and log files (observing user behaviours) |
| de Araujo Lima et al., 2022 [66] | To validate the contents and structure a serious game about vaccine-preventable diseases and immunization contents and heuristics | Educational setting: Experts from nursing college and students from university | Native mobile application | Vaccine-preventable diseases | Development: Heuristic evaluation by users, content evaluation by experts |
| Real et al., 2021 [67] | To assess the usability of the HPV Vaccine: Same Way, Same Day smartphone application (app) on resident clinicians’ perceptions | Educational and healthcare setting: The Academic Pediatric Association and the American Academy of Pediatrics | Native mobile application | Human papillomavirus (HPV) | Development: Usability testing |
| Streuli et al., 2021 [68] | To develop a culturally and linguistically appropriate virtual reality (VR) vaccination education platform | Healthcare and scientific setting: Somali refugees via a community-based organisation, Somali Family Service, and expert advisors | Virtual reality | Paediatric vaccines | Development: Community-based participatory research and co-design |
| **Development and evaluation studies** | | | | | |
| Davies et al., 2015 [69] | To describe the process of the development and report the results of the initial evaluation of a culturally appropriate bilingual app about hepatitis B | Educational and healthcare: Health clinic of a remote community in Arnhem Land. | Mobile or web application (multiple formats available) | Hepatitis B | Development and evaluation: Participatory Action Research |
| Ruiz-López et al., 2019 [70] | To describe the development of a mobile app called Fight HPV, a game-based learning tool | Educational and healthcare setting: Norwegian Women’s Public Health Association, and high school students | Native mobile application | Human papillomavirus (HPV) | Development and evaluation: Iterative design and evaluation via questionnaire |

# **Expanded version of table 3: Tools from Google search and expert suggestion**

| **Name of the gamified tool** | **Link** | **Language** | **Associated Institute or University** | **Country of development** | **Type of disease/vaccine** | **Purpose/objective of the tool** | **Target population** | **Type of gamified digital tool platform** | **Gamification elements (e.g., rewards, role playing, leaderboard, serious game, etc.)** |
| --- | --- | --- | --- | --- | --- | --- | --- | --- | --- |
| Antidote COVID-19 [71] | https://www.eurogamer.net/new-mobile-game-in-collaboration-with-who-raises-covid-19-vaccine-awareness | English | Developed by Psyon Games alongside the UN's World Health Organization, the Vaccine Alliance GAVI, and UNICEF Finland | Finland | COVID-19 | To increase awareness of coronavirus vaccination and other preventative measures | General public | Native mobile application | Reward points |
| The Vaccination Game [72] | https://mrcwimm.itch.io/the-vaccination-game | English | University of Oxford and Goldsmiths, University of London | England | H11N7 and influenza | To illustrate how an effective vaccine can protect individuals and populations from a viral pandemic | General public | Web-based | Serious game |
| Help take down COVID-zilla! [73] | https://www.albertahealthservices.ca/assets/info/ppih/covidzilla/index.html | English | Alberta Health services | Canada | COVID-19 | To defend health, and the health of those around by understanding the importance of protecting themselves against the COVID-19 | Children and youth | Web-based | Role play |
| Just the Vax! [74] | https://media.chop.edu/data/files/vaccine-trivia-game/index.html | English | Children Hospital of Philadelphia | United States | Vaccine preventable disease | To learn about vaccines | High school and College lower division audience | Web-based | Reward points |
| COVID Invaders [75] | https://www.getonedesk.com/covid-invaders | English | One desk | United States | COVID 19 | Not reported | General public | Web-based | Reward points |
| Vax Pack Hero [76] | https://vaxpackhero.com/ | English | Children Hospital of Philadelphia | United States | Vaccine preventable disease | The heroes help players battle 21 different vaccine-preventable diseases in a quest to return to health | ElementarySchool-aged children | Web-based | Reward points and physical trading cards |
| Flu's Clues [77] | https://www.familiesfightingflu.org/virtual-flu-game/ | English & Spanish | University of Iowa | United States | Influenza | To save lives and reduce hospitalizations by protecting children, families, and communities against influenza | Children | Web-based | Certificate of completion for solving the influenza mystery |
| Virus Fighter [78] | https://www.virusfighter.org/ | English | ORION partner Babraham Institute | United Kingdom | COVID-19, Influenza, Ebola, Measles | To engage the public with the science behind vaccinations, infections, and the immune system | Children aged 11-14 (UK school years 7-9) | Web-based | Serious game |
| Immunization411: for preteens and teens’ online training [79] | https://health.mo.gov/living/wellness/immunizations/training/teenspreteens/index.html | English | Missouri Department of Health and Senior Services | United States | Tdap, meningococcal, varicella, HPV and influenza | To train preteens and teens on how people can get diseases from microbes, and how vaccines work in the body to protect and train via interactive game. | Preteen and teens | Web-based | Reward points |
| COVID Chronicles [80] | [https://web.archive.org/web/20220923233038/https://covidchroniclesbc.ca/?v=17](https://web.archive.org/web/20220923233038/https:/covidchroniclesbc.ca/?v=17) | English | The Simon Fraser University and University of British Columbia | Canada | COVID 19 | To show the benefits of getting vaccinated against COVID-19 and to educate further on COVID-19 and vaccines | Young adults | Web-based | Reward points |
| I Boost [81] | https://iboostimmunity.com/ | English | Public Health Association of British Columbia and UNICEF Canada | Canada | Vaccine preventable disease | To educate about immunization and earn a vaccine for people in need | General public | Web-based | Quiz |

#

1. Allocation to a study arm was done according to work schedules, which are often arbitrary. We therefore considered this quasi-randomization. [↑](#footnote-ref-1)
